# The need to address Oral Health concerns for End of Life patients in Long Term Care

**DOI:** 10.1093/geroni/igaf122.3347

**Published:** 2025-12-31

**Authors:** Kadambari Rawal

**Affiliations:** Boston University Henry M. Goldman School of Dental Medicine, Boston, Massachusetts, United States

## Abstract

To understand dental service utilization by frail older adults in a long-term care (LTC) setting in the last 2 years of their life and the characteristics that may be predictors of ‘higher dental service utilization’, a retrospective cohort study was conducted by chart review of LTC patients at 2 sites who had a dental visit in the 2 year period prior to death. Based on the number of dental appointments attended prior to death, the patients were categorized into five groups. A multivariate logistic regression model was created to identify the factors associated with higher dental service utilization. The study found that 84% of patients who died in the study period, utilized on-site dental services in the last 2 years of their life. Approximately 66% had 3 or more dental appointments. Multivariate analysis suggested that Medicaid beneficiaries were more likely to have a higher utilization of dental services than the others. Diagnostic (including emergency & palliative) and preventive procedures were most often performed and denture related procedures (including fabricating new dentures) were commonly performed too. This study showed that a large percentage of frail older adults utilized dental services even in the last years of their lives when given access to these services. As people are living longer and retaining their teeth longer, there is a rising need and subsequent demand for end-of-life dental services. Certain administrative and policy implementation strategies need to be developed to provide dental services to LTC patients in the last years of their lives.

